# A comparison between allogeneic stem cell transplantation from unmanipulated haploidentical and unrelated donors in acute leukemia

**DOI:** 10.1186/s13045-017-0394-2

**Published:** 2017-01-19

**Authors:** Simona Piemontese, F. Ciceri, M. Labopin, W. Arcese, S. Kyrcz-Krzemien, S. Santarone, H. Huang, D. Beelen, N. C. Gorin, C. Craddock, Z. Gulbas, A. Bacigalupo, M. Mohty, A. Nagler, on behalf of the Acute Leukemia Working Party of the European Society for Blood and Marrow Transplantation (EBMT)

**Affiliations:** 1 Hematology and Bone Marrow Transplant Unit, San Raffaele Scientific Institute, Milan, Italy; 2 EBMT ALWP Office, Hospital Saint Antoine, Paris, France; 3 Clinical Hematology and Cellular Therapy Department, Hospital Saint Antoine, APHP, Universite Pierre et Marie Curie, INSERM UMRs 938, Paris, France; 4 Stem Cell Transplant Unit, Fondazione Policlinico Tor Vergata, Tor Vergata University, Rome, Italy; 5 University Department of Hematology and BMT, Medical University of Silesia, Katowice, Poland; 6 Department of Hematology, Ospedale Civile, Pescara, Italy; 7 Bone Marrow Transplantation Center, The First Affiliated Hospital, Zhejiang University School of Medicine, 79 Qingchun Road, Hangzhou, 310003 China; 8 Department of Bone Marrow Transplantation, University Hospital, Essen, Germany; 9 Center for Clinical Hematology, Queen Elizabeth Hospital, Birmingham, UK; 10 Bone Marrow Transplantation Department, Anadolu Medical Center Hospital, Gebze, Turkey; 11 Division of Hematology II, IRCCS, San Martino University Hospital IST, Genoa, Italy; 12 Division of Hematology, Chaim Sheba Medical Center, Tel-Hashomer, Israel

## Abstract

**Background:**

In the absence of a HLA-matched related or matched unrelated donor, allogeneic stem cell transplantation (allo-SCT) from mismatched unrelated donors or haploidentical donors are potential alternatives for patients with acute leukemia with an indication to allo-SCT. The objective of this study was to compare the outcome of allo-SCT from T cell-replete haploidentical (Haplo) versus matched (MUD 10/10) or mismatched unrelated donor at a single HLA-locus (MMUD 9/10) for patients with acute leukemia in remission.

**Methods:**

Two hundred sixty-five adult patients with de novo acute leukemia in first or second remission that received a Haplo-SCT between January 2007 and December 2013 were compared with 2490 patients receiving a MUD 10/10 and 813 receiving a MMUD 9/10. Propensity score weighted analysis was conducted in order to control for disease risk imbalances between the groups.

**Results:**

The weighted 3-year non-relapse mortality and relapse incidence were 29 and 30% for Haplo, 21 and 29% for MUD 10/10, and 29 and 25% for MMUD 9/10, respectively. The weighted 3-year leukemia-free survival (LFS) and overall survival (OS) were 41 and 46% for Haplo, 50 and 56% for MUD 10/10, and 46 and 48% for MMUD 9/10, respectively. Using weighted Cox model, both LFS and OS were significantly higher in transplants from MUD 10/10 compared from those in Haplo but not different between transplants from MMUD 9/10 and Haplo. The type of donor was not significantly associated with neither acute nor chronic graft-versus-host disease.

**Conclusions:**

Patients with acute leukemia in remission have better outcomes if transplanted from a MUD 10/10. We did not find any significant difference in outcome between transplants from MMUD 9/10 and Haplo, suggesting that both can be equally used in the absence of a 10/10 MUD.

**Key point 1:**

Better outcomes using fully (10/10) matched unrelated donor for allo-SCT in acute leukemia in remission.

**Key point 2:**

Similar outcomes after allo-SCT from unmanipulated haploidentical graft or mismatched (9/10) unrelated donor in acute leukemia in remission.

**Electronic supplementary material:**

The online version of this article (doi:10.1186/s13045-017-0394-2) contains supplementary material, which is available to authorized users.

## Background

Allogeneic stem cell transplantation (allo-SCT) represents the only possible cure for most adult acute leukemias (AL). HLA-matched related (MRD) or unrelated donors (MUD) are usually considered the preferable donors, but they are not available for all the patients with an indication for allo-SCT. In the absence of a HLA-matched donor, allo-SCT from mismatched unrelated donors (MMUD), cord blood units (CB), or haploidentical (Haplo) donors are potential alternatives.

A Haplo donor is available for virtually all AL patients, enabling minimal delay and access to repeated stem cell (SC) donations or donor lymphocyte infusions, if needed. These are the main reasons for the increasing numbers of Haplo-SCT for AL in recent years. Unmanipulated (non ex vivo T-depleted) grafts from Haplo donors, in comparison to T-depleted ones, result in lower incidence of serious infections due to faster immune reconstitution and stronger graft versus leukemia effect [1, 2]. In addition, the introduction of more effective regimens for graft-versus-host disease (GvHD) prophylaxis for T-replete Haplo-SCT contributed to reduce GvHD incidence and to increase the use of unmaniplated grafts for the Haplo setting [3–10].

Recently, several reports have shown comparable allo-SCT outcomes between Haplo and historical MRD, MUD, and MMUD series [11–15]. However, these are mostly but one [14] single center studies with limited number of patients [11, 12, 15] in various disease categories and status. For these reasons, we decided to perform a large, registry-based study, using the European Society of Bone Marrow Transplantation (EBMT)-Acute Leukemia Working Party (ALWP) registry, comparing T-replete Haplo-SCT to transplants from MUD and MMUD for AL patients in first or second remission.

## Methods

In order to be included in the study, the patients had to fulfill all the following criteria: age ≥18 years; de novo AL; disease status at transplant: complete remission 1 (CR1) or 2 (CR2); family donor with host/donor number of HLA mismatches ≥2 (Haplo), or MUD 10/10 or MMUD 9/10 (patients and donors should have HLA A, B, C, and DRB1 and DQB1 allelic typing performed); peripheral blood (PB) or bone marrow (BM) or both as source of SC; no ex vivo T cell depletion; and first allo-SCT (previous autologous SCT was allowed). All patients underwent transplantation between January 2007 and December 2013. We were able to verify the inclusion criteria for 265 Haplo-SCT, 2490 MUD 10/10-SCT, and 813 MMUD 9/10-SCT. This was a retrospective multicenter analysis. Data were provided and approved for this study by the ALWP of the EBMT group registry. The EBMT is a non-profit, scientific society representing more than 600 transplant centers mainly in Europe. The EBMT promotes all activity aiming to improve stem cell transplantation or cellular therapy, which includes registering all the activity relating to stem cell transplants. Data are entered, managed, and maintained in a central database with internet access; each EBMT center is represented in this database. There are no restrictions on centers for reporting data, except for those required by the law on patient consent, data confidentiality, and accuracy. Quality control measures included several independent systems: confirmation of validity of the entered data by the reporting team, selective comparison of the survey data with minimum essential data A (MED-A) data sets in the EBMT registry database, cross-checking with the National Registries, and regular in-house and external data audits. Since 1990, patients have provided informed consent authorizing the use of their personal information for research purposes.

### Definitions and statistical analysis

The primary endpoints were leukemia-free survival (LFS) and overall survival (OS). The secondary endpoints were engraftment, acute and chronic GVHD (aGVHD and cGVHD), relapse incidence (RI), non-relapse mortality (NRM), and graft-versus-host relapse-free survival (GRFS) [16]. LFS was defined as time to death or relapse, whichever came first. OS was defined as time to death from all causes. NRM was defined as death without evidence of relapse. Engraftment was defined as the first of three consecutive days with an absolute neutrophil count > 0.5 × 10^9^/L. Acute GVHD was graded according to the modified Seattle-Glucksberg criteria [17] and cGVHD according to the revised Seattle criteria [18].

We used propensity scores (PS) weighting to control for pre-treatment imbalances on observed variables. The following factors were included in the PS model: patient age, time from diagnosis to transplantation, year of transplant, diagnosis (AML versus ALL), status at transplant (CR1 versus CR2), cytogenetics group, donor/patient CMV serology, conditioning (RIC versus MAC), and sex matching (female donor to male recipient versus other). The estimation of propensity score was performed using generalized boosted models [19].

As the study question was whether Haplo could replace 10/10 or 9/10 MUD, we weighted the groups receiving either MUD 10/10- and MMUD 9/10-HSCT to match the characteristics of patients receiving Haplo-SCT, by estimating the average treatment effect among the treated (ATT); Haplo-HSCT being the treated group. The ATT weights equal one for Haplo-HSCT, and it equals the ratio of the propensity score to one minus the propensity score in the two UD-HSCT groups. Therefore, UD patients that were significantly different from average haplo-grafted patients had a low contribution in the comparisons. We checked the balance between the groups looking to ATT weighted means. Then, we used pairwise ATTs to fit the weighted Kaplan-Meier and Cox models separately for Haplo- versus MUD 10/10-HSCT and Haplo- versus MMUD 9/10-HSCT.

The type I error rate was fixed at 0.05 for determination of factors associated with time to event. Analyses were performed using the R statistical software version 3.2.3 (R Development Core Team, Vienna, Austria); propensity score analysis was performed using the *mnps* function of the Twang package and weighted analyses using the survey package [20].

## Results

### Patients’ and donors’ characteristics

Patient’s and host/donors’ characteristics are showed in Table 1. Regimens, stem cell source, and GvHD prophylaxis are described in Table 2. Anti-thymocyte globulin (ATG) was used for in vivo T cell depletion in 120/265 Haplo (45%), 1457/2490 (59%) 10/10 MUD, and 550/813 (82%) 9/10 MMUD. Campath was used in 6/265 Haplo (1%), 302/2490 (12%) MUD, and 124/813 (18%) MMUD. PT-Cy was used in 107/265 Haplo (40%), 27/2490 (1%) MUD, and 12/813 (1%) MMUD. Among patients receiving a MMUD 9/10, 204 (25%) were mismatched in locus A, 115 (14%) in locus B, 275 (34%) in locus C, 62 (8%) in locus DRB1, and 157 (19%) in locus DQB1. Allelic typing for locus A, B, C, DRB1, and DQB1 was available for all patients and donors.

**Table 1:** Patients’ and host/donor characteristics

	Haplo	MUD10/10	MMUD 9/10	MUD 10/10 vs Haplo	MMUD 9/10 vs Haplo
No.	265	2490	813		
Follow-up	34.2 (3–84)	35.7 (1–103)	35.8 (1–102)	0.93	0.72
Age	42.8 (18–75)	47.0 (18–76)	44.8 (18–71)	<10–3	0.017
Year of Tx	2011 (07–13)	2010 (07–13)	2010 (07–13)	<10–3	<10–3
Diagnosis to transplant	260 days (82–5784)	196 days (71–5793)	223d (73–4452)	<10–3	0.016
Diagnosis					
AML	176	1645	510		
	66%	66%	63%		
ALL	89	845	303	0.90	0.28
	34%	34%	37%		
Disease status					
CR1	159	1901	580		
	60%	76%	71%		
CR2	106	589	233	<10–3	0.001
	40%	24%	29%		
Patient sex					
Male	155	1344	442		
	58%	54%	54%		
Female	110	1146	371	0.16	0.24
	42%	46%	46%		
Donor sex					
Male	139	1782	490		
	52%	72%	61%		
Female	126	690	312	<10–3	0.013
	48%	28%	39%		
Female to male					
No	191	2186	659		
	72%	88%	82%		
Yes	74	286	143	<10–3	<10–3
	28%	12%	18%		
CMV neg to neg					
No	225	1673	577		
	86%	69%	73%		
Yes	36	754	212	<10–3	<10–3
	14%	31%	27%		
Cytogenetics					
Good	29	261	68		
	11%	10%	8%		
Intermediate	168	1398	446		
	63%	56%	55%		
Poor	68	831	299	0.04	0.004
	26%	33%	37%		

See text for abbreviations. Cytogenetic risk at diagnosis for AML: good t(8;21), inv16 or t(16;16); poor monosomy/deletion 5 or 7, abnormalities 11q23, complex karyotype (≥3 abnormalities); intermediate all the others. Cytogenetic risk at diagnosis for ALL: adverse t(9;22) or t(4;11), intermediate all the others

**Table 2:** Conditioning regimens, stem cell source, and in vivo T cell depletion

No.	Haplo	MUD10/10	MMUD 9/10	MUD 10/10 vs Haplo	MMUD 9/10 vs Haplo
	265	2490	813		
Conditioning					
MAC	138	1460	489		
	52%	59%	60%		
RIC	127	1018	322	0.03	0.02
	48%	41%	40%		
Stem cell source					
BM	141	473	141	<10–4	<10–4
	53%	19%	17%		
PB	124	2017	672		
	47%	81%	83%		
In vivo T cell depletion					
No	38	720	137	<10–4	<10–4
	15%	29%	17%		
ATG	120	1743	664		
	45%	70%	82%		
Pt-Cy	107	27	12		
	40%	1%	1%		

See text for abbreviations

### Engraftment

The 30-day CI of engraftment was 95% (92–97%) for Haplo, 97% (93–98%) for MUD 10/10, and 92% (88–95%) for MMUD 9/10. In weighted Cox model, CI of engraftment resulted to be lower in Haplo versus MUD 10/10 (*p* = 0.015) but not different between Haplo and MMUD 9/10 (*p* = 0.62).

### Non-relapse mortality, acute and chronic GvHD

**Fig. 1:**
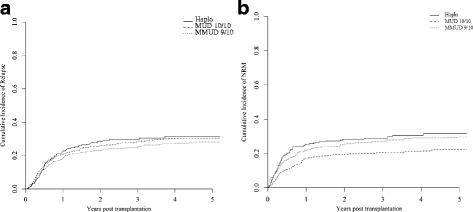
Weighted CI of relapse and non-relapse mortality according to donor type. **a** Weighted CI of relapse. **b** Weighted CI of NRM

The weighted CI of NRM at 3 years was 29% (23–34%), 21% (15–26%), and 29% (23–35%) for Haplo , MUD 10/10, and MMUD 9/10 (Fig. 1a), respectively. In weighted Cox model, NRM was lower in MUD 10/10 as compared in Haplo, but not different from that in MMUD 9/10 (Table 3). The percentage of grades II–IV aGvHD was 28% (22–33%), 25% (18–31%), and 27% (21–33%) for Haplo, MUD 10/10, and MMUD 9/10, respectively. The frequency of grades III–IV aGvHD was 10% (6–13%) for Haplo, 7% (3–10%) for MUD 10/10, and 11% (6–15%) for MMUD 9/10. The 3-year CI of overall cGvHD and extensive cGvHD was 34% (28–40%) and 15% (10–20%), 40% (33–47%) and 22% (15–28%), and 33% (26–39%) and 18% (12–23%) for Haplo, MUD 10/10, and MMUD 9/10, respectively. Neither grades II–IV aGvHD nor chronic GvHD incidences differed between Haplo versus MUD 10/10 and Haplo versus MMUD 9/10 in weighted Cox analysis (Table 3).

**Table 3:** Weighted Cox model for NRM, RI, LFS, OS, and GRFS

3-year	MUD 10/10 vs Haplo	*p*	MMUD 9/10 vs Haplo	*p*
	HR (95%CI)		HR (95%CI)	
Day 30 PMN engraftment	0.845 (0.740-0.968)	0.015	0.961 (0.819-1.127)	0.623
II–IV aGvHD	0.8877 (0.6777–1.163)	0.387	1.0433 (0.7716–1.411)	0.783
III–IV aGvHD	0.673 (0.4334–1.045)	0.078	1.184 (0.7221–1.942)	0.5027
cGVHD all	1.0763 (0.8474–1.367)	0.547	0.9555 (0.7264–1.257)	0.745
Extensive cGvHD	1.483 (0.9890–2.224)	0.057	1.288 (0.8342–1.990)	0.253
NRM	0.6363 (0.4838–0.8369)	0.0012	0.9905 (0.7009–1.3997)	0.957
RI	0.8624 (0.6668–1.115)	0.259	0.8426 (0.6312–1.125)	0.245
LFS	0.7487 (0.6229–0.8998)	0.002	0.9164 (0.7296–1.1511)	0.45
OS	0.7074 (0.5824–0.8591)	0.0005	0.9300 (0.7302–1.1844)	0.56
GRFS	0.8804 (0.7433–1.043)	0.14	1.0313 (0.8370–1.271)	0.772

See text for abbreviations

### Relapse incidence

The weighted CI of relapse at 3 years was 30% (24–35%), 29% (22–35%), and 25% (19–31%) for Haplo, MUD 10/10, and MMUD 9/10, respectively (Fig. 1b).The type of donor was not a predictive factor for relapse in weighted Cox model (Table 3).

### LFS, OS, and GRFS

**Fig. 2:**
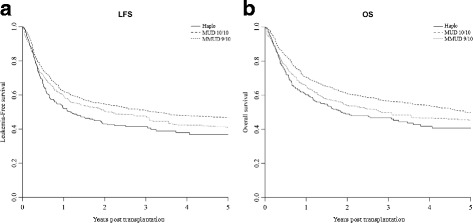
Weighted probability of leukemia-free survival and overall survival according to donor type. **a** Weighted probability of leukemia-free survival. **b** Weighted probability of overall survival

The median follow-up among survivors was 34 (range, 3–84) months for Haplo, 36 (range, 1–103) for MUD 10/10, and 36 (range, 1–102) for MMUD 9/10, respectively. The weighted probability of LFS at 3 years was 41% (35–48%), 50% (43–58%), and 46% (39–53%) for Haplo, 10/10 MUD, and 9/10 MMUD, respectively (Fig. 2).The weighted probability of 3-year OS was 46% (40–53%), 56% (49–64%), and 48% (41–56%) (Fig. 2).The 3-year GRFS was 33% (28–40%), 36% (29–44%), and 34% (28–41%) for Haplo, 10/10 MUD, and 9/10 MMUD, respectively (Fig. 2). In the weighted Cox analysis, LFS and OS resulted to be better for MUD 10/10 compared with Haplo, but no statistical difference comparing Haplo with MUD 9/10. No statistical differences were found in GRFS according to the type of donor in the weighted Cox analysis (Table 3).

## Discussion

In the absence of a MRD for allo-SCT in acute leukemia, the ideal donor still remains to be determined. The current report represents a large registry study comparing the outcome between transplants from Haplo and MUD 10/10 or MMUD 9/10 for adult patients with de novo AL in remission.

As a recent ALWP-EBMT survey on unmanipulated haploidentical transplantation in AL showed a lower incidence of aGvHD in patients receiving PT-Cy but no significant statistical differences in either LFS or OS [21], in our study we included all the non T-depleted Haplo-SCT registered in the EBMT database.

Previous reports described worse outcomes after MMUD in comparison to MUD [22–26], and this finding was in accordance to our preliminary data and to a recent ALWP publication [27].

We did not find any differences in terms of LSF and OS in the 813 MMUD-SCT according to HLA-DQ mismatch in univariate analysis (*p* = 0.35). Therefore, we decided to compare separately MUD 10/10 and MMUD 9/10 transplants with Haplo.

The 30-day CI of engraftment proved to be lower in Haplo than in MUD 10/10 but not different between Haplo and MMUD 9/10. In addition to the type of donor, the number and type of chemotherapy cycles pre-transplant and/or early post-transplant infections, in particular viral, could have had an impact on differences in engraftment. A lower incidence of engraftment in Haplo compared in UD was previously reported in both reduced intensity [14] and myeloablative conditioning transplants [15].

Notably, we did not find any difference in the incidence of either aGvHD > II or cGvHD according to donor type in line with similar comparisons in literature. This was true also for grades III–IV aGvHD and extensive cGvHD. In the recent report comparing Haplo using PT-Cy to UD from the Center for International Blood and Marrow Transplant Research (CIBMTR), the authors describe a lower incidence of acute and cGvHD in Haplo on univariate analysis [14], but most of the Haplo were performed using BM as stem cell source, and most of the UD using PB without receiving any in vivo T cell depletion. Of note, they did not find any difference in the 3-year cGvHD incidence according to donor type when analyzing only patients receiving BM. The role of stem cell source as risk factor for cGvHD after Haplo-SCT remains to be determined. While some reports do not show any difference in terms of cGvHD between PB and BM in haploidentical transplantations using PT-Cy [28, 29], a recent comparison from CIBMTR concludes for a high incidence of cGvHD using PB instead of BM [30]. Notable, in the context of UD, cGvHD has been shown to be higher using PBSC [31] especially if not using in vivo T cell depletion [32].

The 3-year NRM was higher using Haplo (29%) compared with MUD 10/10 (21%) but identical compared with MMUD (29%), in contrast with other reports on similar comparisons where the authors did not find any difference [11–15]. This higher NRM in Haplo compared in MUD 10/10 could be in part due to the use of in vivo T cell depletion other than PT-Cy in the 45% of our Haplo [33]. In addition to the type of in vivo T cell depletion, also, differences in conditioning regimens could explain our finding [14]. Patients’ comorbidities, mostly unknown in restrospective study, could also have influenced NRM. The 3-year CI of relapse was not different between the three groups.

The lower NRM in MUD 10/10 resulted in both a higher 3-year LFS and OS compared in Haplo but no difference in GRFS. No differences in either OS, LFS, or GRFS were observed between Haplo and MMUD 9/10. Analyzing separately patients with AML and ALL, we found no differences of GRFS in Haplo compared in MUD 10/10 and compared in MMUD 9/10, higher LFS and OS in MUD 10/10 compared in Haplo but no differences between Haplo and MMUD 9/10 in terms of LFS and OS (Additional file 1).

Based on these results, we can assert that patients with acute leukemia in remission showed better outcomes if transplanted from a MUD 10/10. We did not find any significant difference in outcome between MMUD 9/10 and Haplo, suggesting that both can be equally used in the absence of a MUD 10/10 and that other factors, such as urgency of transplant and center expertise should dictate the choice between these two alternative donor sources.

Ongoing and future prospective clinical studies, including transplants from CBU, will ultimately be required to determine the best alternative donor for adult AL patients who lack an HLA-matched sibling one.

## Additional files

Additional file 1: Table AML and ALL. (DOCX 18 kb)
Additional file 2: Participating centers’ Table. (DOCX 37 kb)

## Abbreviations

AL: Acute leukemia
ALL: Acute lymphoblastic leukemia
AML: Acute myeloid leukemia
ATG: Anti-thymocyte globulin
ATT: Average treatment effect among the treated
BM: Bone marrow
CB: Cord blood
CMV: Cytomegalovirus
CR: Complete remission
GRFS: Graft-versus-host relapse-free survival
GVHD: Graft-versus-host disease (acute aGvHD or chronic cGvHD)
HAPLO: Haploidentical donor
LFS: Leukemia-free survival
MAC: Myeloablative conditioning regimen
MMUD: Mismatched unrelated donor
MRD: Matched related donor
MUD: Matched unrelated donor
NRM: Non-relapse mortality
OS: Overall survival
PB: Peripheral blood
PMN: Polymorphonuclear cells
PS: Propensity score
PT-Cy: Post-transplant cyclophosphamide
RI: Relapse incidence
RIC: Reduced intensity conditioning regimen
SC: Stem cell
SCT: Stem cells transplantation
TX: Transplant
