# Supplementary material for: A comparison between allogeneic stem cell transplantation from unmanipulated haploidentical and unrelated donors in acute leukemia
Source: J Hematol Oncol. 2017 Jan 19;10:24. doi: 10.1186/s13045-017-0394-2 (PMC5248464; doi:10.1186/s13045-017-0394-2)
Supplement: Additional file 1: — Table AML and ALL. (DOCX 18 kb) [file 13045_2017_394_MOESM1_ESM.docx]

| **AML** | **Weighted Kaplan-Meier estimator** | | | **Weighted Cox analysis** |  |  |  |
| --- | --- | --- | --- | --- | --- | --- | --- |
|  |  |  |  | MUD 10/10 vs HAPLO |  | MMUD 9/10 vs HAPLO |  |
| **3 y outcome** | HAPLO | MUD 10/10 | MMUD 9/10 | HR (95% CI) | P | HR (95% CI) | P |
| day30 engraftment | 94 (89-96)% | 98 (93-99)% | 93 (87-96)% | 1.379 ( 1.169 - 1.627) | **0.0001** | 1.282 ( 1.047 - 1.569) | **0.016** |
| aGVHD II-IV | 25 (18-31)% | 23 (16-30)% | 24 (17-31)% | 0.9454 (0.6754 - 1.323) | 0.744 | 1.0893 (0.7375 - 1.609) | 0.667 |
| aGVHD III-IV | 10 (6-15)% | 6 (2-10)% | 9 (4-13)% | 0.5779 (0.3404 - 0.981) | **0.042** | 0.8840 (0.4933 - 1.584) | 0.679 |
| cGVHD | 31 (23-38)% | 40 (30-48)% | 33 (24-40)% | 1.213 (0.8977 - 1.638) | 0.209 | 1.133 (0.8032 - 1.599) | 0.476 |
| ext.cGVHD | 15 (8-20)% | 20 (12-27)% | 16 (9-23)% | 1.414 (0.8596 - 2.324) | 0.173 | 1.241 (0.7212 - 2.135) | 0.435 |
| NRM | 28 (21-34)% | 18 (11-25)% | 25 (18-32)% | 0.5762 (0.4121 -0.8056) | **0.0013** | 0.9449 (0.6032 -1.4803) | 0.804 |
| RI | 27 (20-0.34)% | 29 (20-37)% | 28 (19-35)% | 0.9440 (0.6824 - 1.306) | 0.728 | 0.9864 (0.6903 - 1.410) | 0.940 |
| LFS | 45 (38-53)% | 53 (45-63)% | 47 (39-56)% | 0.7563 (0.599 -0.9549) | **0.02** | 0.9650 (0.725 -1.2845) | 0.807 |
| OS | 52 (44-60)% | 58 (50-68)% | 49 (41-59)% | 0.746 (0.5826 -0.9553) | **0.02** | 1.014 (0.7494 -1.3731) | 0.926 |
| GRFS | 38 (31-46)% | 40 (32-50)% | 34 (26-43)% | 0.8974 (0.7205 - 1.118) | 0.334 | 1.1095 (0.8544 - 1.441) | 0.436 |
|  |  |  |  |  |  |  |  |
|  |  |  |  |  |  |  |  |
|  |  |  |  |  |  |  |  |
| **ALL** | **Weighted Kaplan-Meier estimator** | | | **Weighted Cox analysis** |  |  |  |
|  |  |  |  | MUD 10/10 vs HAPLO |  | MMUD 9/10 vs HAPLO |  |
| **3 y outcome** | HAPLO | MUD 10/10 | MMUD 9/10 | HR (95% CI) | P | HR (95% CI) | P |
| day30 engraftment | 100% | 95 (86-98)% | 92 (81-96)% | 0.8385 (0.6680 -1.0525) | 0.129 | 0.6695 (0.5165 -0.8679) | **0.002** |
| aGVHD II-IV | 34 (24-44)% | 30 (17-40)% | 33 (20-43)% | 0.8217 (0.5241 - 1.288) | 0.392 | 0.9875 (0.6141 - 1.588) | 0.959 |
| aGVHD III-IV | 8 (2-14)% | 8 (1-14)% | 15 (6-23)% | 0.9257 (0.4130 - 2.075) | 0.851 | 1.9792 (0.8056 - 4.863) | 0.137 |
| cGVHD | 41 (29-51)% | 41 (27-52)% | 33 (20-44)% | 0.8353 (0.5655 - 1.234) | 0.366 | 0.6599 (0.4213 - 1.034) | 0.070 |
| ext.cGVHD | 16 (6-25)% | 26 (13-37)% | 20 (9-30)% | 1.593 (0.8088 - 3.139) | 0.178 | 1.382 (0.6714 - 2.845) | 0.380 |
| NRM | 31 (21-40)% | 26 (14-36)% | 37 (24-48)% | 0.5762 (0.4121 -0.8056) | 0.175 | 0.9449 (0.6032 -1.4803) | 0.795 |
| RI | 35 (24-45)% | 29 (17-40)% | 20 (9-29)% | 0.7344 (0.4818 - 1.119) | 0.151 | 0.6113 (0.3715 - 1.006) | 0.053 |
| LFS | 34 (25-46)% | 45 (34-60)% | 43 (32-57)% | 0.7323 (0.5454 -0.9832) | **0.038** | 0.8375 (0.5753 -1.2193) | 0.355 |
| OS | 36 (27-48)% | 52 (41-67)% | 46 (35-60)% | 0.6396 (0.4685 - 0.873) | **0.005** | 0.8001 (0.5345 - 1.198) | 0.278 |
| GRFS | 25 (17-36)% | 28 (18-42)% | 34 (24-49)% | 0.8472 (0.6550 - 1.096) | 0.207 | 0.9091 (0.6427 - 1.286) | 0.590 |
