# Supplementary material for: A comparison between allogeneic stem cell transplantation from unmanipulated haploidentical and unrelated donors in acute leukemia
Source: J Hematol Oncol. 2017 Jan 19;10:24. doi: 10.1186/s13045-017-0394-2 (PMC5248464; doi:10.1186/s13045-017-0394-2)
Supplement: Additional file 2: — Participating centers’ Table. (DOCX 37 kb) [file 13045_2017_394_MOESM2_ESM.docx]

**Additional file 1**

EBMT participating centres:

| **MUD 10/10** |  | **MMUD 9/10** |  | **Haplos** |  |
| --- | --- | --- | --- | --- | --- |
| Centers | pts | Centers | pts | Centers | pts |
| 515 Helsinki [Univ Central H] | 81 | 387 Birmingham [Queen Elizabeth] | 25 | 756 Rome [Tor Vergata] | 31 |
| 267 Pessac [H Haut-Leveque] | 68 | 207 Paris [St Louis] | 22 | 813 Milano [S Raffaele] | 25 |
| 246 Rotterdam [Erasmus ` den Hoed] | 67 | 515 Helsinki [Univ Central H] | 22 | 217 Genova [S Martino] | 16 |
| 387 Birmingham [Queen Elizabeth] | 63 | 212 Stockholm [Univ H] | 17 | 919 Antalya [Medical Park H] | 16 |
| 277 Lille [H Claude Huriez] | 61 | 230 Marseille [Paoli Calmettes] | 17 | 248 Pescara [Osp Civile] | 15 |
| 207 Paris [St Louis] | 60 | 277 Lille [H Claude Huriez] | 17 | 401 Hangzhou [Univ H] | 11 |
| 717 Nottingham [City H] | 55 | 671 Lyon [H E Herriot] | 17 | 587 Reggio_Calabria [Centro Trapianti] | 11 |
| 230 Marseille [Paoli Calmettes] | 49 | 234 Brussels [St. Luc] | 16 | 440 Kocaeli [Anadolu] | 9 |
| 253 Nantes [Hotel Dieu] | 49 | 246 Rotterdam [Erasmus ` den Hoed] | 16 | 513 Munich [Kl Grosshadern] | 9 |
| 676 Vandoeuvre_Les_Nancy [H d`Enfants] | 44 | 270 Grenoble [H A Michallon] | 16 | 931 Suzhou [First Soochow] | 9 |
| 235 Oslo [Rikshospitalet] | 43 | 676 Vandoeuvre_Les_Nancy [H d`Enfants] | 16 | 231 Torino [S. Giovanni (CTO)] | 8 |
| 270 Grenoble [H A Michallon] | 43 | 235 Oslo [Rikshospitalet] | 14 | 230 Marseille [Paoli Calmettes] | 7 |
| 202 Basel [202] | 42 | 253 Nantes [Hotel Dieu] | 14 | 763 London [Kings College H] | 6 |
| 624 Toulouse [H Purpan] | 37 | 717 Nottingham [City H] | 14 | 544 Monza [Osp S Gerardo] | 5 |
| 283 Lund [Univ H] | 36 | 566 Cambridge [Addenbrookes H] | 13 | 354 Milano [Trapianto Midollo Osseo] | 4 |
| 212 Stockholm [Univ H] | 34 | 785 Homburg [Univ Saarland] | 13 | 775 Paris [St Antoine] | 4 |
| 311 Wiesbaden [Kl Diagnostik] | 34 | 931 Suzhou [First Soochow] | 13 | 788 Ancona [Umberto I] | 4 |
| 650 Angers [CHRU] | 34 | 202 Basel [202] | 12 | 789 Avellino [S G Moscati] | 4 |
| 209 Leuven [Univ H] | 33 | 242 Santander [Valdecilla] | 12 | 202 Basel [202] | 3 |
| 259 Essen [Univ H] | 32 | 672 Strasbourg [H Hautepierre] | 12 | 259 Essen [Univ H] | 3 |
| 672 Strasbourg [H Hautepierre] | 31 | 926 Montpellier [University] | 12 | 397 Riyadh [King Faisal] | 3 |
| 926 Montpellier [University] | 31 | 257 Dublin [St James] | 10 | 658 Bergamo [Ospedale, ematol] | 3 |
| 233 Besancon [H Jean Minjoz] | 29 | 267 Pessac [H Haut-Leveque] | 10 | 808 Dresden [Universitaets Kl] | 3 |
| 671 Lyon [H E Herriot] | 29 | 283 Lund [Univ H] | 10 | 204 Ulm [Medizin Kl / Polikl] | 2 |
| 661 Rennes [H Sud/Pontchaillou] | 28 | 209 Leuven [Univ H] | 9 | 250 Saint_Etienne [St Etienne] | 2 |
| 565 Maastricht [Univ H] | 27 | 218 London [Royal Marsden] | 9 | 253 Nantes [Hotel Dieu] | 2 |
| 566 Cambridge [Addenbrookes H] | 27 | 624 Toulouse [H Purpan] | 9 | 261 Geneva [261] | 2 |
| 225 Turku [University] | 26 | 813 Milano [S Raffaele] | 9 | 287 Rome [S Camillo - Forlanini] | 2 |
| 251 Caen [Hopital, Hematol] | 26 | 239 Utrecht [University] | 8 | 524 Heidelberg [Medizinishce Kl] | 2 |
| 264 Poitiers [H La Miletrie] | 26 | 251 Caen [Hopital, Hematol] | 8 | 561 Thessaloniki [G Papanicolaou G H] | 2 |
| 556 Budapest [National Med Ctr] | 26 | 264 Poitiers [H La Miletrie] | 8 | 614 Hamburg [Univ H] | 2 |
| 242 Santander [Valdecilla] | 23 | 273 Clermont-Ferrand [Jean Perrin] | 8 | 717 Nottingham [City H] | 2 |
| 704 Southampton [General H] | 23 | 544 Monza [Osp S Gerardo] | 8 | 722 Palma_De_Mallorca [Son Dureta] | 2 |
| 718 Pilsen [Charles Univ H] | 23 | 565 Maastricht [Univ H] | 8 | 731 Umeå [Univ H] | 2 |
| 931 Suzhou [First Soochow] | 23 | 658 Bergamo [Ospedale, ematol] | 8 | 169 Ankara [Gazi Univ] | 1 |
| 273 Clermont-Ferrand [Jean Perrin] | 22 | 718 Pilsen [Charles Univ H] | 8 | 205 London [Hammersmith] | 1 |
| 725 St._Petersburg [Pavlov Med Univ] | 21 | 740 Linköping [Univ H] | 8 | 209 Leuven [Univ H] | 1 |
| 726 Liege [University] | 21 | 252 Creteil [H Mondor Hematol] | 7 | 211 Sao_Paulo [H Sirio-Libanes] | 1 |
| 731 Umeå [Univ H] | 21 | 295 Hannover [Medical Univ] | 7 | 214 Barcelona [H Clinic] | 1 |
| 785 Homburg [Univ Saarland] | 21 | 713 Leicester [Royal Infirmary] | 7 | 215 Brussels [Jules Bordet] | 1 |
| 807 Berlin [Charite Univ] | 21 | 808 Dresden [Universitaets Kl] | 7 | 223 Tübingen [UnivTubingen] | 1 |
| 941 Rouen [Becquerel] | 21 | 152 Augsburg [Zentra Kl] | 6 | 256 Kiel [UKSH] | 1 |
| 677 Katowice [Silesian Med Acad] | 20 | 203 Leiden [Univ H] | 6 | 260 Barcelona [SCreu i S Pau] | 1 |
| 234 Brussels [St. Luc] | 19 | 206 Copenhagen [Rigshospitalet] | 6 | 281 Patras [Univ H] | 1 |
| 659 Brest [C.H.R.U Brest] | 19 | 259 Essen [Univ H] | 6 | 346 Sofia [Queen Johanna] | 1 |
| 813 Milano [S Raffaele] | 19 | 286 Pavia [S Matteo] | 6 | 387 Birmingham [Queen Elizabeth] | 1 |
| 252 Creteil [H Mondor Hematol] | 18 | 289 Goeteborg [Sahlgrenska Univ H] | 6 | 392 Palermo [Osp V Cervello] | 1 |
| 644 Vilnius [Santariskiy Kl] | 18 | 304 Firenze [Careggi-Meyer] | 6 | 515 Helsinki [Univ Central H] | 1 |
| 206 Copenhagen [Rigshospitalet] | 17 | 311 Wiesbaden [Kl Diagnostik] | 6 | 557 Pavia [S Matteo] | 1 |
| 239 Utrecht [University] | 17 | 704 Southampton [General H] | 6 | 584 Barcelona [V d`Hebron Adults] | 1 |
| 289 Goeteborg [Sahlgrenska Univ H] | 17 | 731 Umeå [Univ H] | 6 | 589 Adana [Baskent Univ] | 1 |
| 295 Hannover [Medical Univ] | 17 | 778 Sheffield [Royal Hallamshire] | 6 | 633 Teheran [Shariati] | 1 |
| 523 Nice [H de l`ARCHET I] | 17 | 941 Rouen [Becquerel] | 6 | 640 Ljubljana [Univ Med Ctr] | 1 |
| 546 Groningen [Univ H] | 17 | 996 Antwerp_Edegem [UZA] | 6 | 652 Tricase_(Lecce) [C Panico] | 1 |
| 614 Hamburg [Univ H] | 17 | 141 Brescia [Civili, Adulti] | 5 | 656 Prague [Ist Hematology] | 1 |
| 513 Munich [Kl Grosshadern] | 16 | 225 Turku [University] | 5 | 692 Palermo [La Maddalena] | 1 |
| 740 Linköping [Univ H] | 16 | 232 Rome [Emat, `La Sapienza`] | 5 | 705 Udine [Univ H] | 1 |
| 756 Rome [Tor Vergata] | 16 | 258 Jerusalem [Univ Hadassah] | 5 | 718 Pilsen [Charles Univ H] | 1 |
| 810 Freiburg [University] | 16 | 294 Milano [Osp Niguarda] | 5 | 725 St._Petersburg [Pavlov Med Univ] | 1 |
| 996 Antwerp_Edegem [UZA] | 16 | 601 Manchester [Royal Infirmary] | 5 | 744 Gent [Univ H] | 1 |
| 152 Augsburg [Zentra Kl] | 15 | 650 Angers [CHRU] | 5 | 754 Tel-Hashomer [Univ Adults] | 1 |
| 215 Brussels [Jules Bordet] | 15 | 725 St._Petersburg [Pavlov Med Univ] | 5 | 766 Napoli [Federico II] | 1 |
| 218 London [Royal Marsden] | 15 | 726 Liege [University] | 5 | 773 Liverpool [Alder Hey] | 1 |
| 231 Torino [S. Giovanni (CTO)] | 15 | 756 Rome [Tor Vergata] | 5 | 791 Cagliari [Osp A Businco] | 1 |
| 658 Bergamo [Ospedale, ematol] | 15 | 214 Barcelona [H Clinic] | 4 | 794 Perugia [Monteluce] | 1 |
| 257 Dublin [St James] | 14 | 233 Besancon [H Jean Minjoz] | 4 | 810 Freiburg [University] | 1 |
| 775 Paris [St Antoine] | 14 | 556 Budapest [National Med Ctr] | 4 | 811 Cagliari [R Binaghi] | 1 |
| 977 Limoges [CHRU] | 14 | 597 Brno [Univ H] | 4 | 819 Madrid [H G Marañón] | 1 |
| 746 Tartu [Univ H] | 12 | 610 Bratislava [Univ H] | 4 | Total | 265 |

| **MUD 10/10** |  | **MMUD 9/10** |  |
| --- | --- | --- | --- |
| Centers | pts | Centers | pts |
| 778 Sheffield [Royal Hallamshire] | 12 | 614 Hamburg [Univ H] | 4 |
| 203 Leiden [Univ H] | 11 | 623 Verona [Policlinico] | 4 |
| 204 Ulm [Medizin Kl / Polikl] | 11 | 659 Brest [C.H.R.U Brest] | 4 |
| 248 Pescara [Osp Civile] | 11 | 769 Sevilla [Virgen del Rocio] | 4 |
| 250 Saint_Etienne [St Etienne] | 11 | 775 Paris [St Antoine] | 4 |
| 240 Bologna [S Orsola-Malpighi] | 10 | 807 Berlin [Charite Univ] | 4 |
| 389 Leipzig [Univ, Haemat/Oncol] | 10 | 810 Freiburg [University] | 4 |
| 597 Brno [Univ H] | 10 | 161 Tel_Aviv [Sourasky] | 3 |
| 625 Nürnberg [Klinikum] | 10 | 215 Brussels [Jules Bordet] | 3 |
| 656 Prague [Ist Hematology] | 10 | 223 Tübingen [UnivTubingen] | 3 |
| 161 Tel_Aviv [Sourasky] | 9 | 224 London [UCL] | 3 |
| 260 Barcelona [SCreu i S Pau] | 9 | 227 Vienna [Medizinische Univ] | 3 |
| 386 Bristol [Royal H Sick Children] | 9 | 240 Bologna [S Orsola-Malpighi] | 3 |
| 665 Clamart [H Percy] | 9 | 261 Geneva [261] | 3 |
| 224 London [UCL] | 8 | 303 Cardiff [Univ Wales] | 3 |
| 261 Geneva [261] | 8 | 523 Nice [H de l`ARCHET I] | 3 |
| 544 Monza [Osp S Gerardo] | 8 | 533 Jena [Friedrich-Schiller] | 3 |
| 727 Salamanca [H Clinico] | 8 | 546 Groningen [Univ H] | 3 |
| 808 Dresden [Universitaets Kl] | 8 | 561 Thessaloniki [G Papanicolaou G H] | 3 |
| 208 Zürich [208] | 7 | 598 San_Sebastian [H Aranzazu] | 3 |
| 232 Rome [Emat, `La Sapienza`] | 7 | 656 Prague [Ist Hematology] | 3 |
| 302 Zagreb [Univ H Rebro] | 7 | 677 Katowice [Silesian Med Acad] | 3 |
| 534 Cologne [Univ, Medicine] | 7 | 825 Alessandria [SS Antonio e Biagio] | 3 |
| 552 Gottingen [Universitaetskl] | 7 | 145 Stuttgart [Robert Bosch Kh] | 2 |
| 623 Verona [Policlinico] | 7 | 160 Paris [H Necker] | 2 |
| 729 Hradec_Králové [Charles Univ H, Hematol] | 7 | 204 Ulm [Medizin Kl / Polikl] | 2 |
| 256 Kiel [UKSH] | 6 | 205 London [Hammersmith] | 2 |
| 290 Karlsruhe [Klinikum] | 6 | 208 Zürich [208] | 2 |
| 345 Haifa [Rambam MCH] | 6 | 231 Torino [S. Giovanni (CTO)] | 2 |
| 409 Petach-Tikva [Beilinson H] | 6 | 265 Milano [Osp Maggiore] | 2 |
| 640 Ljubljana [Univ Med Ctr] | 6 | 300 Lisboa [Inst Oncologia] | 2 |
| 145 Stuttgart [Robert Bosch Kh] | 5 | 386 Bristol [Royal H Sick Children] | 2 |
| 223 Tübingen [UnivTubingen] | 5 | 389 Leipzig [Univ, Haemat/Oncol] | 2 |
| 401 Hangzhou [Univ H] | 5 | 513 Munich [Kl Grosshadern] | 2 |
| 524 Heidelberg [Medizinishce Kl] | 5 | 524 Heidelberg [Medizinishce Kl] | 2 |
| 574 Olomouc [Univ H] | 5 | 530 Greifswald [Ernst-Moritz-Arndt] | 2 |
| 588 Amsterdam [VU Univ Med Ctr] | 5 | 602 Bremen [Kl Bremen-Mitte] | 2 |
| 693 Warsaw [Inst Haematology] | 5 | 606 Cuneo [S Croce e Carle] | 2 |
| 699 Wroclaw [Medical Acad] | 5 | 613 Barcelona [H Trias i Pujol] | 2 |
| 769 Sevilla [Virgen del Rocio] | 5 | 617 Ankara [Ibni Sina H] | 2 |
| 786 Mainz [Johannes-Gutenberg] | 5 | 693 Warsaw [Inst Haematology] | 2 |
| 825 Alessandria [SS Antonio e Biagio] | 5 | 727 Salamanca [H Clinico] | 2 |
| 160 Paris [H Necker] | 4 | 780 Manchester [Christie] | 2 |
| 205 London [Hammersmith] | 4 | 786 Mainz [Johannes-Gutenberg] | 2 |
| 214 Barcelona [H Clinic] | 4 | 788 Ancona [Umberto I] | 2 |
| 255 Oxford [Radcliffe H] | 4 | 798 Christchurch [Canterbury Health] | 2 |
| 258 Jerusalem [Univ Hadassah] | 4 | 977 Limoges [CHRU] | 2 |
| 286 Pavia [S Matteo] | 4 | 153 Hamburg [AK St Georg] | 1 |
| 303 Cardiff [Univ Wales] | 4 | 169 Ankara [Gazi Univ] | 1 |
| 308 Graz [Medical Univ] | 4 | 244 Glasgow [Royal Infirmary] | 1 |
| 533 Jena [Friedrich-Schiller] | 4 | 248 Pescara [Osp Civile] | 1 |
| 538 Wroclaw [Ctr Cell Transpl] | 4 | 250 Saint_Etienne [St Etienne] | 1 |
| 602 Bremen [Kl Bremen-Mitte] | 4 | 256 Kiel [UKSH] | 1 |
| 610 Bratislava [Univ H] | 4 | 260 Barcelona [SCreu i S Pau] | 1 |
| 642 Oviedo [H Covadonga] | 4 | 266 Uppsala [Univ H] | 1 |
| 649 Bari [Univ Studi] | 4 | 271 Innsbruck [Univ H] | 1 |
| 792 Catania [Osp Ferrarotto] | 4 | 281 Patras [Univ H] | 1 |
| 930 Moscow [NRC Haem.] | 4 | 290 Karlsruhe [Klinikum] | 1 |
| 954 Warsaw [Central] | 4 | 291 Porto [Inst Oncologia] | 1 |
| 141 Brescia [Civili, Adulti] | 3 | 308 Graz [Medical Univ] | 1 |
| 153 Hamburg [AK St Georg] | 3 | 321 Siena [Le Scotte] | 1 |
| 169 Ankara [Gazi Univ] | 3 | 332 Taranto [Osp Nord] | 1 |
| 227 Vienna [Medizinische Univ] | 3 | 345 Haifa [Rambam MCH] | 1 |
| 237 Nijmegen [St Radboud] | 3 | 397 Riyadh [King Faisal] | 1 |
| 276 Newcastle-Upon-Tyne [Royal Victoria] | 3 | 401 Hangzhou [Univ H] | 1 |
| 281 Patras [Univ H] | 3 | 409 Petach-Tikva [Beilinson H] | 1 |
| 291 Porto [Inst Oncologia] | 3 | 529 Pesaro [Osp, Transplant Ctr] | 1 |
| 294 Milano [Osp Niguarda] | 3 | 543 Modena [Policlinico] | 1 |
| 300 Lisboa [Inst Oncologia] | 3 | 570 Santiago_De_Compostela [H Clin Univ] | 1 |
| 304 Firenze [Careggi-Meyer] | 3 | 588 Amsterdam [VU Univ Med Ctr] | 1 |
| 305 Torino [Regina Margherita] | 3 | 594 Linz [Elisabethinen H] | 1 |
| 354 Milano [Trapianto Midollo Osseo] | 3 | 625 Nürnberg [Klinikum] | 1 |
| 530 Greifswald [Ernst-Moritz-Arndt] | 3 | 640 Ljubljana [Univ Med Ctr] | 1 |
| 630 Brussels [Univ H] | 3 | 644 Vilnius [Santariskiy Kl] | 1 |
| 680 Münster [University] | 3 | 649 Bari [Univ Studi] | 1 |
| 713 Leicester [Royal Infirmary] | 3 | 661 Rennes [H Sud/Pontchaillou] | 1 |
| 744 Gent [Univ H] | 3 | 680 Münster [University] | 1 |
| 754 Tel-Hashomer [Univ Adults] | 3 | 692 Palermo [La Maddalena] | 1 |
| 759 Barcelona [H Univ Bellvitge] | 3 | 729 Hradec_Králové [Charles Univ H, Hematol] | 1 |
| 780 Manchester [Christie] | 3 | 744 Gent [Univ H] | 1 |
| 798 Christchurch [Canterbury Health] | 3 | 746 Tartu [Univ H] | 1 |
| 811 Cagliari [R Binaghi] | 3 | 763 London [Kings College H] | 1 |
| 819 Madrid [H G Marañón] | 3 | 768 London [S Bartholomew`s] | 1 |
| 244 Glasgow [Royal Infirmary] | 2 | 787 Regensburg [University] | 1 |
| 265 Milano [Osp Maggiore] | 2 | 791 Cagliari [Osp A Businco] | 1 |
| 287 Rome [S Camillo - Forlanini] | 2 | 792 Catania [Osp Ferrarotto] | 1 |
| 323 Murcia [V Arrixaca] | 2 | 795 Pisa [Az Osp Univ] | 1 |
| 390 Düsseldorf [Heinrich Heine Univ] | 2 | 799 Gdansk [Medical U] | 1 |
| 392 Palermo [Osp V Cervello] | 2 | 809 Erlangen [University] | 1 |
| 526 San_Giovanni_Rotondo [IRCCS] | 2 | 819 Madrid [H G Marañón] | 1 |
| 561 Thessaloniki [G Papanicolaou G H] | 2 | 954 Warsaw [Central] | 1 |
| 570 Santiago_De_Compostela [H Clin Univ] | 2 | 994 Istanbul [Nightingale] | 1 |
| 577 Pamplona [H de Navarra] | 2 | Total | 813 |

| **MUD 10/10** |  |
| --- | --- |
| Centers | pts |
| 584 Barcelona [V d`Hebron Adults] | 2 |
| 594 Linz [Elisabethinen H] | 2 |
| 606 Cuneo [S Croce e Carle] | 2 |
| 645 Marburg [Philipps Univ] | 2 |
| 652 Tricase_(Lecce) [C Panico] | 2 |
| 705 Udine [Univ H] | 2 |
| 749 Oldenburg [Klinikum] | 2 |
| 772 Cape_Town [Constantiaberg] | 2 |
| 791 Cagliari [Osp A Businco] | 2 |
| 955 Amiens [H Sud] | 2 |
| 119 Ascoli_Piceno [Osp Mazzoni] | 1 |
| 211 Sao_Paulo [H Sirio-Libanes] | 1 |
| 236 Madrid [Princesa] | 1 |
| 254 Leeds [St James] | 1 |
| 262 Paris [Pitie-Salpetrriere] | 1 |
| 266 Uppsala [Univ H] | 1 |
| 282 Valencia [H Clinico] | 1 |
| 299 Bolzano [Osp S Maurizio] | 1 |
| 356 Salzburg [LKA, Oncology] | 1 |
| 367 Lübeck [Universitaet] | 1 |
| 369 Beirut [American Univ] | 1 |
| 397 Riyadh [King Faisal] | 1 |
| 452 Prague [U H Motol] | 1 |
| 501 Liverpool [Royal Univ H] | 1 |
| 506 Brugge [AZ Sint-Jan] | 1 |
| 529 Pesaro [Osp, Transplant Ctr] | 1 |
| 543 Modena [Policlinico] | 1 |
| 557 Pavia [S Matteo] | 1 |
| 590 Berlin [Benjamin Franklin] | 1 |
| 591 Minsk [Ctr Paediatric] | 1 |
| 592 Idar-Oberstein [Kl Knochenmarktr] | 1 |
| 601 Manchester [Royal Infirmary] | 1 |
| 613 Barcelona [H Trias i Pujol] | 1 |
| 617 Ankara [Ibni Sina H] | 1 |
| 634 Aarhus [Univ, Hematol] | 1 |
| 666 Villejuif [Gustave Roussy] | 1 |
| 692 Palermo [La Maddalena] | 1 |
| 710 Perth [Royal H] | 1 |
| 712 Würzburg [Medizinische Kl II] | 1 |
| 730 Poznan [K Marcinkowski Univ] | 1 |
| 752 Athens [Agia Sophia] | 1 |
| 760 Istanbul [Tip Fakueltesi] | 1 |
| 763 London [Kings College H] | 1 |
| 764 Bydgoszcz [Collegium Medicum] | 1 |
| 768 London [S Bartholomew`s] | 1 |
| 787 Regensburg [University] | 1 |
| 788 Ancona [Umberto I] | 1 |
| 806 Lyon [H Debrousse] | 1 |
| 809 Erlangen [University] | 1 |
| 823 Plymouth [Derriford H] | 1 |
| 970 Flensburg [St Franziskus] | 1 |
| 994 Istanbul [Nightingale] | 1 |
| Total | 2490 |
